# Lower regional grey matter in alcohol use disorders: evidence from a voxel-based meta-analysis

**DOI:** 10.1186/s12888-021-03244-9

**Published:** 2021-05-11

**Authors:** Lei Li, Hua Yu, Yihao Liu, Ya-jing Meng, Xiao-jing Li, Chengcheng Zhang, Sugai Liang, Ming-li Li, Wanjun Guo, QiangWang, Wei Deng, Xiaohong Ma, Jeremy Coid, Tao Li

**Affiliations:** 1 Mental Health Center, West China Hospital of Sichuan University, Chengdu, Sichuan People’s Republic of China; 2 Psychiatric Laboratory, State Key Laboratory of Biotherapy, West China Hospital, Sichuan University, Chengdu, Sichuan People’s Republic of China; 3 Brain Research Center, West China Hospital of Sichuan University, Chengdu, China; 4 Department of Psychology, College of Life and Environmental Science, University of Exeter, Exeter, UK

**Keywords:** Alcohol use disorders, Abstinence, Grey matter, Meta-analysis, Voxel-based morphometry

## Abstract

**Background:**

Previous research using whole-brain neuroimaging techniques has revealed structural differences of grey matter (GM) in alcohol use disorder (AUD) patients. However, some of the findings diverge from other neuroimaging studies and require further replication. The quantity of relevant research has, thus far, been limited and the association between GM and abstinence duration of AUD patients has not yet been systematically reviewed.

**Methods:**

The present research conducted a meta-analysis of voxel-based GM studies in AUD patients published before Jan 2021. The study utilised a whole brain-based d-mapping approach to explore GM changes in AUD patients, and further analysed the relationship between GM deficits, abstinence duration and individual differences.

**Results:**

The current research included 23 studies with a sample size of 846 AUD patients and 878 controls. The d-mapping approach identified lower GM in brain regions including the right cingulate gyrus, right insula and left middle frontal gyrus in AUD patients compared to controls. Meta-regression analyses found increasing GM atrophy in the right insula associated with the longer mean abstinence duration of the samples in the studies in our analysis. GM atrophy was also found positively correlated with the mean age of the samples in the right insula, and positively correlated with male ratio in the left middle frontal gyrus.

**Conclusions:**

GM atrophy was found in the cingulate gyrus and insula in AUD patients. These findings align with published meta-analyses, suggesting they are potential deficits for AUD patients. Abstinence duration, age and gender also affect GM atrophy in AUD patients. This research provides some evidence of the underlying neuroanatomical nature of AUD.

**Supplementary Information:**

The online version contains supplementary material available at 10.1186/s12888-021-03244-9.

## Background

The literature has long framed alcohol use disorders (AUD) as chronic brain diseases [1]. The brain disease model of addiction states that GM atrophy involves in the reward circuit, as well as brain regions relevant to decision-making [2, 3]. More importantly, this abnormality of the reward circuit and decision making would negatively reinforce to craving, which leads to substance abuse eventually being compulsive [4]. The disease model is supported by numerous neuroimaging studies using structural magnetic resonance imaging (MRI) by analysing voxel-based morphometry (VBM). However, the pathology of this disease model requires clarification.

VBM analysis by MRI techniques has provided evidence of GM deficits in cortical areas, including the prefrontal cortex (PFC) [5], anterior cingulate gyrus (ACC) [6], parietal cortex [7], and in subcortical brain regions, including the hippocampus [8], the thalamus, the nucleus accumbens [9] and the amygdala [10] in individuals with AUD. Those brain regions are believed to be involved with decision-making and reward processing. Previous meta-analysis and reviews have suggested that GM is lower in the cingulate gyrus, striatum and insula in AUD patients compared to control samples [11, 12], whereas observed changes in the PFC, dorsal lateral prefrontal cortex, left thalamus and right hippocampus remain inconsistent.

One of the possibilities is that the sample gender ratio may contribute to individual differences in GM volume. It has been reported in some previous studies that females are more vulnerable to the effects of alcohol on GM than males [13]. However, other studies reported that males with AUD had lower GM in the thalamus and putamen compared to their nondrinking peers, whereas females with AUD had greater GM in the thalamus and putamen [14]. The gender ratio in Xiao et al. [11] was not examined, which could have affected findings.

Another possibility posits that the samples age and the age at onset could moderate the rate of GM atrophy. For example, adolescent brains are especially susceptible to the effects of alcohol [15]. It was reported that those who started drinking before the age of 16 are more likely to develop alcohol dependence than those who started after 21 [16]. Alternatively, Sullivan et al. [17] reported a controversial age-alcoholism interaction showing that older individuals with alcohol dependence obtain greater deficits than controls because older brains are more susceptible to alcohol, regardless of the duration of alcohol dependence. Thayer et al. [18] reported that samples under the age of 25 would suffer serious GM atrophy and different brain regions seemed to suffer the effect, where increase of GM atrophy in the left lateral orbitofrontal cortex was correlated with age in the samples over 25 years old. On the other hand, Xiao et al. [11] did not find evidence for any effect of age. The results from these previous studies were therefore inconsistent.

Apart from age and gender, the duration of time after abstinence from alcohol could also potentially affect GM. Recent research reported a sustained compensatory effect on GM in the cingulate gyrus and insular regions, that is, no more significant GM atrophy was found after abstinence from alcohol, suggesting that abstinence from alcohol potentially allows for structural recovery in GM in AUD patients [19]. However, this effect diminished in the pre-cuneus that the GM atrophy reappeared with a longer abstinence duration. In other words, individuals with alcohol dependence could experience corresponding atrophy in GM after abstaining from alcohol. Mann et al. reviewed similar cases in AUD patients [20]. Therefore, the current study will perform meta-regression analyses of abstinence duration for AUD to provide comparable replication.

The current research includes newly published studies on AUD (within the past 21 years) and performed an anisotropic effect size-signed differential mapping (AES-SDM) for neuroimaging studies. A meta-analysis using the SDM toolbox identified the most consistent brain changes in space with the coordinate information reported in previous studies. We aimed to establish the most consistent brain structural abnormalities of AUD, using all published, whole-brain structural MRI studies that do not bias findings to a priori hypothesised regions. Then, the impact of AUD abstinence and other individual differences on the GM morphology with the latest studies and a larger sample size of AUD patients was examined. From the literature reviewed above, we hypothesised that, firstly, the GM of the AUD group would differ significantly from the control group, and these differences would show consistency with previously published meta results; Secondly, mean abstinence duration in the studies and individual parameters of the samples, such as mean age and gender ratio would have effects on the GM atrophy.

## Methods

### Data source

A systematic and comprehensive search strategy was used to collect studies in PubMed (https://www.ncbi.nlm.nih.gov/pubmed/), CENTER (Cochrane Library) (https://www.cochrane.org/), Embase (www.embase.com), and Google Scholar (http://scholar.google.com/) from Jan 2000 to Jan 2021. DSM-V combines diagnostic criteria for abuse and dependence into a unitary diagnostic category of AUD. This study utilised a combination of the following keywords: (1) voxel-based morphometry; VBM; morphometry; volumetry; grey matter; or structural MRI and (2) alcohol dependent; alcoholism; alcohol abuse or alcohol use disorder.

### Inclusion/exclusion criteria

The reference lists of identified studies and relevant theoretical reviews were then manually checked for additional studies.

The inclusion criteria: (1) subjects with alcohol abuse disorder, alcohol dependence disorder or alcohol use disorder that met the DSM-IV-TR or International Statistical Classification of Diseases and Related Health Problems-10th Revision (ICD-10) diagnostic criteria; (2) use of VBM to analyse the GM differences in patients with AUD and control subjects; (3) results of whole-brain GM alterations reported in MNI space; (4) participants aged ≥18, and all participants provided informed consent; (5) thresholds for significance corrected for multiple comparisons; (6) peer reviewed studies; and (7) articles published in English (for quality check purpose).

**Fig. 1:**
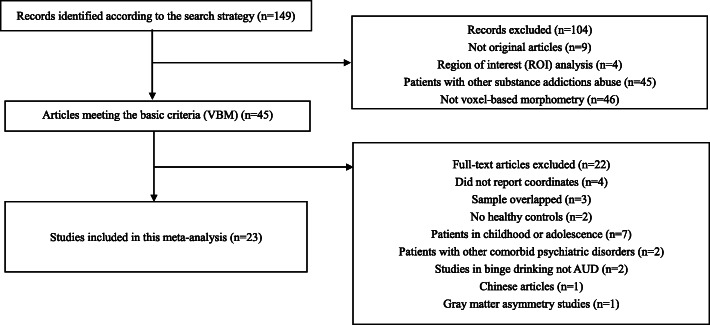
Flowchart of the selection of VBM studies in patients with AUD for meta-analysis

The exclusion criteria: (1) studies used a region-of-interest (ROI) or seed voxel–based analysis only; (2) studies included participants aged < 18 years old; (3) studies analysed white matter changes or cortical thickness only; (4) research material comprised review articles, theoretical papers or animal experimental studies; (5) Chinese articles; (6) studies included patients with other comorbid psychiatric disorders; and (7) original coordinates were not reported, and the author did not respond to email inquiries (Fig. 1).

To evaluate the perceived studies’ quality, the following criteria was applied: (1) group matching, (2) method of alcohol use diagnosis, (3) group matching on drug use levels, (4) samples size, (5) method used to collect alcohol use history, (6) the use of GM volume or density covariates, (7) MRI machines, smooth kernels, corrected level. Each criterion was independently assessed by two independent reviewers who scored a numerical value from 0 to 5 to evaluate quality. The sum was used in a meta-regression to check the effect of quality.

### AES-SDM

Regional differences in GM between patients with AUD and controls were analysed with AES-SDM (AES-SDM; http://www.sdmproject.com/) [21]. In brief, the main steps of AES-SDM were as follows: Firstly, we extracted peak coordinates to recreate txt file in the included studies, the effect size, such as P- or Z- values for clusters were transformed into t-statistics by SDM online conversion utilities (https://www.sdmproject.com/utilities/?show=Statistics). A total of 23 txt files were generated. Secondly, we imported 23 txt files into the SDM software and recreated a sdm_table. Finally, we conducted pre-processing, mean analysis, sensitivity analysis, heterogeneity analyses, meta-regression analysis of mean age, the proportion of males, abstinence duration (days), age at onset and duration of dependence. The studies were given different weights based on number of participants and quality of research, weighted by the square root of the sample size and quality of each study [21].

A whole-brain voxel-based jackknife sensitivity analysis assessed the reproducibility of the results. The analysis was repeated 23 times, with each iteration leaving out a different study. Conclusions can be drawn if differences for a brain region remain significant in more than 75% of the sensitivity analyses. Variables assessed with the meta-regression analysis included mean age, the percentage of males and abstinence duration as well as age at onset and duration of dependence (less than half of the 23 studies provided this information). Heterogeneity analyses determined if there were significant, unexplained differences between studies. Funnel plots and Egger tests identified conflicting studies and publication bias [22].

## Results

### Demographic and clinical characteristics of patients with AUD and controls

The current research included 23 studies in the meta-analysis based on the search strategy. In total, there were 846 patients with AUD (male = 658; female = 188; mean age range: 22.95–53.6 years) and 878 controls (male = 679; female = 199; mean age range: 24.63–53.7 years). Table 1 illustrated the demographic information. No differences were found in age, gender or education between AUD and control groups in each study.

**Table 1:** Demographic and clinical characteristics of the AUD participants in the study in the meta-analysis

Study	AUD patients					Healthy controls		Methodology			
	No. (male)	Mean age, yr	Diagnosis	Diagnosis criteria	Abstinence or not (abstinence duration)	No. (male)	Mean age, yr	MRI scanner	Smooth kernel	Volume or density	Corrected level
Jang et al. (2007)	20 (20)	43.6 (6)	Alcohol-dependence	DSM-IV	Yes (7.8 days)^2^	20 (20)	44.5 (7.4)	3.0 T	8	density	FDR, *p* < 0.05
Mechtcheriakov et al.(2007)	22 (14)	53.6	Alcohol addiction	ICD-10	Yes (10 days)^2^	22 (14)	53.7	1.5 T	10	volume	FDR, *p* < 0.05
Chanraud et al. (2007)	26 (26)	47.7 (7.1)	Alcohol-dependence	DSM-IV	Yes (3 weeks)^2^	24 (24)	45 (6.7)	1.5 T	8	volume	FDR *p* < 0.005
Chanraud et al. (2009)	24 (24)	47.8 (7.7)	Alcohol-dependence	DSM-IV	Yes (3 weeks)^2^	24 (24)	45 (5.6)	1.5 T	Ns	volume	Not mention
Demirakca et al. (2011)	50 (27)	45 (10.1)	Alcohol-dependence	DSM-IV &ICD-10	Yes(16.5 days)^2^	66 (34)	44.1 (10.3)	1.5 T	8	volume	FWE, *p* < 0.05
Rando et al.(2011)	55(35)	38.2	alcohol-dependent	DSM-IV	Yes(1 month)^1^	50(28)	31.14	Ns	8	volume	FWE,*p* < 0.025
Grodin et al.(2012)	37(21)	40.2(9.2)	alcoholics	DSM-IV	Yes(21.5 days)^2^	69(47)	36.6(1.1)	1.5 T	3	volume	*p* < 0.01
van Holst et al. (2012)	36 (36)	43.2 (11.03)	AUD	DSM-IV-TR	Yes (18 days)^2^	54 (54)	35.3 (10.1)	3.0 T	8	volume	FDR, *p* < 0.05
Howell et al. (2013)	19 (7)	22.95 (3.41)	Alcohol Abuse	AADC	Yes (24 h)^2^	19 (7)	24.63 (4.40)	3.0 T	10	volume	K = 19, *p* < 0.05
Charlet et al.(2014)	40(30)	44.9(11.4)	alcohol-dependent	DSM-IV	Yes(11.4 days)^2^	40(30)	44.1(12.0)	3.0 T	8	volume	FWE,*p* < 0.05
Asensio et al.(2015)	24(24)	35.62(4.81)	alcohol abuse	DSM-IV	Yes (3 days)^2^	24(24)	31.91(9.3)	1.5 T	8	volume	*p* < 0.05
Padula et al.(2015)	14(6)	43.21 (9.13)	Alcohol-dependent	DSM-IV	Yes (1 month)^2^	14(5)	37.57 (12.08)	3.0	10	volume	Not mention
Romanczuk-Seiferth(2015)	15(15)	45.40 (10.15)	alcohol-dependent	ICD-10&DSM-IV	Yes(42 days)^2^	17(17)	37.41 (11.76)	Ns	8	volume	Not mention
Wiers et al.(2015)	22(22)	42.14(6.20)	alcohol dependence	DSM-IV	Yes(48 days)^2^	21(21)	41.95(6.4)	3.0 T	10	volume	FWE,*p* < 0.005
Wang et al.(2016)	20(20)	43.95(8.17)	alcohol dependence	DSM-IV	Yes (1 month)^2^	20(20)	40.5(6.30)	3.0 T	8	volume	FWE,*P* < 0.05
Guggenmos et al.(2017)	119(101)	45(10.7)	AD	DSM-IV-TR&ICD-10	Yes(22.8 days)^2^	97(81)	43.7(10.8)	3.0 T	8	volume	FWE, *p* < 0.05
Bach et al.(2017)	74(55)	46.55(10.02)	alcohol-dependent	DSM	Yes(2.5 days)^2^	43(27)	46.49(9.2)	3.0 T	8	volume	FWE,*p* < 0.05
Zois et al.(2017)	95(71)	45.9(9.9)	AD	DSM-IV	Yes(11.7 days)^2^	87(71)	45.9(10.6)	3.0 T	8	volume	FWE,*p* < 0.05
Wu et al.(2018)	9(5)	47(11.2)	alcohol-dependent	Not mention	Yes(14 days)^2^	22(13)	44.9(9.5)	3.0 T	8	volume	FWE,*p* < 0.05
Wang et al.(2018)	21(21)	45.95 (7.07)	alcohol dependence	DSM-IV	Yes(50.58 days)^2^	33(33)	42.88 (6.05)	3.0 T	8	volume	FWE,*p* < 0.05
Li et al.(2019)	20(17)	49(12)	alcohol addiction	ICD-10	Not mention	20(17)	49(12)	3.0 T	8	volume	Not mention
Bach et al.(2020)	62(48)	25.8(4.4)	alcohol-dependent	DSM-IV	Yes(11.77 days)^2^	74(58)	44.12(9.6)	3.0 T	15	volume	FWE,*p* < 0.05
Galandra et al.(2020)	22(13)	45.59 (7.99)	Alcohol Use Disorder	DSM-V	Yes(28 days)^2^	18(10)	44.83 (8.86)	3.0 T	8	density	FWE,*p* < 0.05

Note: AADC, National Institute on Alcoholism and Alcohol Abuse diagnostic criteria; AUD, alcohol use disorders, AD, alcohol dependence and those patients would be alcohol abuse or dependence either. Ns, not mention

^1^ Abstinence = 4 weeks of negative urine screens; ^2^Abstinence = the moment an individual stops using the substance

### Regional GM differences

**Fig. 2:**
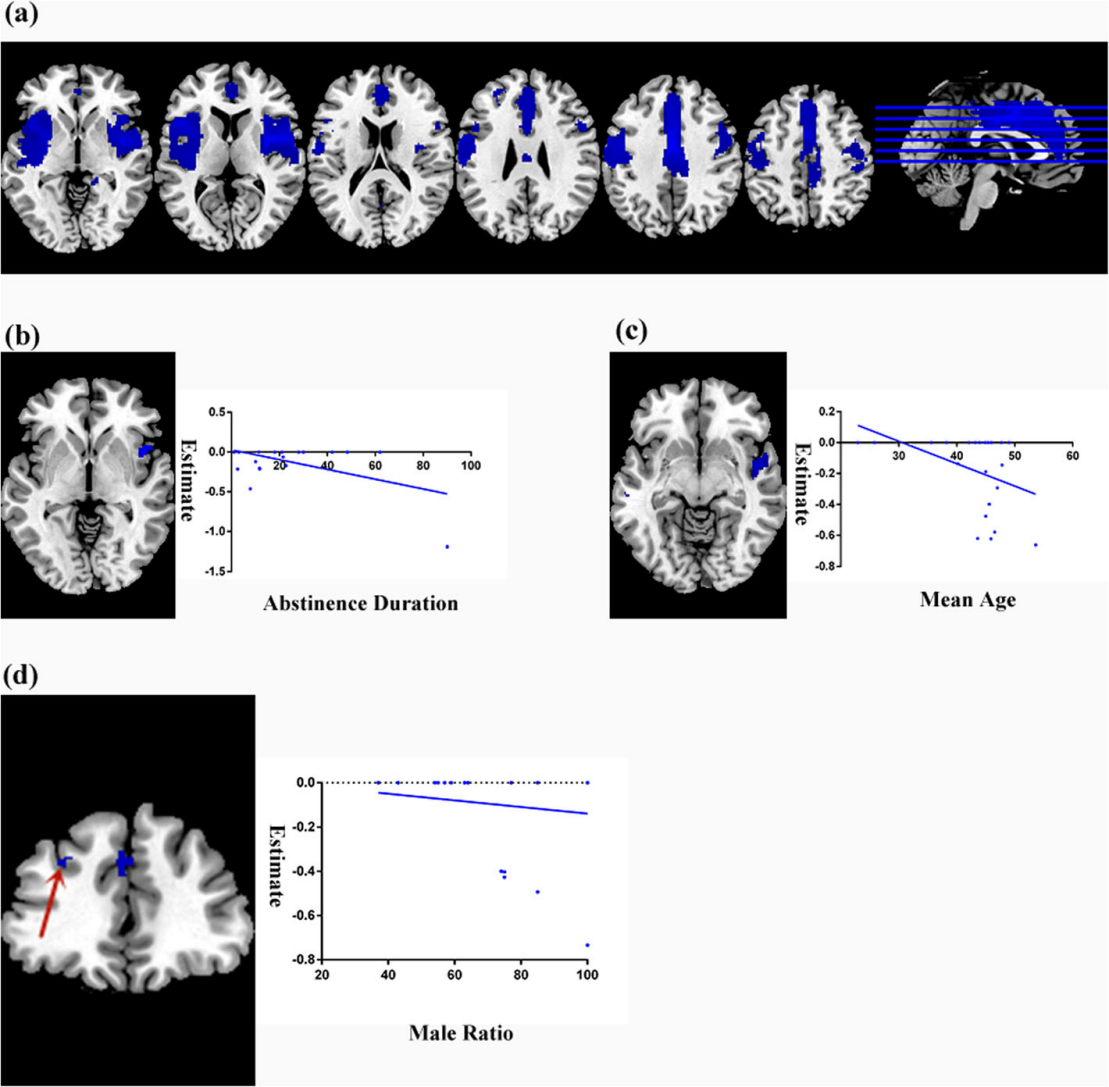
Meta-analysis results. **a** Regions showed lower GM in AUD patients than controls. **b** Meta-regression analysis indicated that GM in the right insula was significantly negatively associated with duration of abstinence in AUD patients. **c** Meta-regression analysis indicated that GM in the right insula was significantly negatively associated with mean age in AUD patients. **d** Meta-regression analysis showed that GM in the left middle frontal gyrus was significantly negatively associated with male ratio in AUD patients. Blue colour represents GM reduction

The pooled AES-SDM meta-analysis map revealed significant lower GM in the right cingulate gyrus, right insula and left middle frontal gyrus in AUD patients compared to controls (Fig. 2a). Table 2 shows the peak coordinates and the cluster breakdown. No brain areas with increased GM were observed.

**Table 2:** Lower grey matter in patients with AUD compared with controls in the meta-analysis

Anatomical regions	MNI coordinates x, y, z	SDM value	*p* value	Number of voxels	Breakdown
AUD < controls					
Right median cingulate / paracingulate gyri (BA24)	2,6,42	−4.438	~ 0	249	Right median cingulate / paracingulate gyri, BA24 Left median cingulate / paracingulate gyri, BA24
Right insula, BA 48	40,0,4	−4.581	0.000010*****	3641	Right insula, BA 48 Right rolandic operculum, BA 48 Right inferior frontal gyrus, opercular part, BA 44,48 Right precentral gyrus, BA 4,6
Left middle frontal gyrus, BA 46	−28,42,30	−3.573	0.00015****	106	Left middle frontal gyrus, BA 46 Left middle frontal gyrus

AUD = Alcohol abuse disorders; MNI = Montreal Neurological Institute; SDM = signed differential mapping; BA = Brodmann area; *****p < 0.00001; ****p < 0.0001

### Sensitivity and heterogeneity analyses

Whole-brain jackknife sensitivity analysis of the findings showed that the main results were highly robust. As shown in Table 3, the whole-brain jackknife sensitivity analysis revealed that lower GM in the right insula and right cingulate gyrus were highly replicable, as this finding was preserved when each study was removed. Lower GM in the left middle frontal gyrus remained significant in 22 out of 23 combinations. Finally, heterogeneity analysis results were reflected by the funnel plot and Egger tests, and the funnel plots did not reveal any publication bias. (see supplementary materials, Fig. S1).

**Table 3:** Regions of grey matter differences in AUD patients compared with controls for sensitivity analyses

MNI coordinate	SDM-Z	P value	Voxels	Clusters	Jackknife sensitivity analysis
2,6,42	−4.438	~ 0	249	right cingulate	23/23
40,0,4	−4.581	0.000010*****	3641	Right insula	23/23
−28,42,30	−3.573	0.00015****	106	Left middle frontal gyrus	22/23

AUD = Alcohol abuse disorders; MNI = Montreal Neurological Institute; SDM = signed differential mapping; BA = Brodmann area; *****p < 0.00001; ****p < 0.0001

### Meta-regression analysis of abstinence, age and gender

The results of the meta-regression analysis on abstinence duration from the AUD patients revealed an increasing of atrophy in the right insula (MNI coordinate: 44, 10, − 4; 55 peak voxels; SDM z = − 4.371; *p* = 0.0015) with the longer mean abstinence duration of the samples (Fig. 2b). GM in right insula (MNI coordinate: 54, − 2, 8; 532 peak voxels; SDM z = − 4.243; *p* = ~.000) yields a significant negative correlation with the average age of the samples (Fig. 2c). There was a negative association between the male ratio of the studies and GM in the left middle frontal gyrus (MNI coordinate: − 28, 38, 32; 11 peak voxels; SDM z = − 2.682; *p* = 0.002) (Fig. 2d). Moreover, exploratory meta-regression analysis suggests that the age at onset and duration of dependence in AUD patients was negatively associated with GM volume (supplementary materials, Fig. S2).

## Discussion

The current quantitative meta-analysis of VBM studies included 23 original studies which demonstrated GM reduction in AUD patients with a large samples size. The results showed lower GM in the right cingulate gyrus, right insula and the left middle frontal gyrus in AUD patients compared with controls, which were highly replicated with previously published meta-analysis. Jackknife sensitivity analyses showed that these results were consistent and robust [12]. Then, we found that GM atrophy increased with longer mean duration of abstinence of the samples in the studies. Finally, we found that GM atrophy increased with mean age and the age onset in the samples, and the male ratio of the samples were more sensitive to the harmful effects of alcohol consumption.

### GM atrophy in the ACC, insula and middle frontal gyrus

The results revealed a significant GM atrophy in the ACC and right insula in AUD patients compared to controls, which aligns with previously published reviews [11, 12]. The ACC is related to a range of cognitive functions, including impulse control and assessment of responses and behaviour [23, 24]. Craving for alcohol in AUD patients is related to abnormal brain circuits projected from the ACC to the nucleus accumbens, and GM volume changes may form the structural basis of this [25]. In the healthy controls, inhibitory control was found to be associated with activation in the ACC [26]. However, in healthy adults with a family history of alcoholism, higher impulsivity was associated with lower activation in the ACC [27]. Compared to health controls, smaller GM volume in the ACC was found in alcohol users with higher impulsivity [28]. However, the current studies included in our meta-analysis are not longitudinally designed, and we cannot make a firm conclusion about the causal relationship between GM atrophy and alcohol consumption.

Apart from the ACC, the insula is also an important hub for transmitting salient information to the prefrontal cortices involved in decision-making [29], to the limbic system involved in emotional responses [30] and to the brain reward system via the ventral striatum involved in substance abuse [31]. Functional brain imaging studies have revealed a correlation between insula activity and cravings for alcohol and drugs [32]. The current study reported consistent results: GM in the right insula was lower in the AUD group compared with the controls, replicating previous results.

The current study also demonstrated brain changes not observed in the two previous meta-analyses [11, 12]. The GM in the left middle frontal gyrus in AUD patients were lower than that in the controls. The middle frontal gyrus is thought to play an important role in cognitive functions, including working memory capacity and fluid intelligence [33]. Yang et al. [12] found that shrinkage of GM in the left middle frontal gyrus correlates to lifetime alcohol consumption. Previous studies reported that the prefrontal function of alcohol users experiences gradually damage, which may be related to an increase in alcohol consumption, which is likely to promote increased craving and weaken behavioural control, thereby contributing to further alcohol consumption [34, 35].

We replicated previous meta-analysis findings in the ACC and right insula, which identified deficits of GM in the ACC and insula as potential neuroanatomical diagnostic biomarkers in AUD patients. However, GM loss in parietal, temporal and subcortical brain regions was not replicated. One possible explanation is that, with our increased sample size, the observed group effect disappeared, suggesting that GM deficits of these brain areas do not represent stable markers of AUD. Another possible explanation posits that, in the current study, we failed to control whether patients had received effective treatment as drugs may influence these brain regions. A previous animal study in rats found that naltrexone treatment during early abstinence resulted in subtle brain changes potentially distinguishable from non-treated abstinent brains, suggesting the existence of an intermediate state associated with brain recovery from alcohol exposure induced by medication [36]. Future studies should consider drug treatment effects on GM volume.

### The persistent of GM atrophy after abstinence

The current meta-regression analysis found that the right insula shows persistent GM reduction in AUD patients with increasing abstinence duration of the samples, which is comparable with findings in previous studies [37, 38]. However, other studies reported diverse results [39], where the frontal, temporal and insular regions remain no different from controls after abstinence from opioids. One key difference may be the treatment received by patients. The current study, as well as previous studies [37, 38], failed to control whether samples have received any treatment, what type of treatment or the intensity, because this information was unavailable. According to Wollman and colleagues’ 2017 study [39], all participants received drug substitution therapy (methadone) to achieve abstinence and no GM atrophy was observed post treatment. Therefore, the abstinence in the current study should not represent as recovery but only abstinence from alcohol consumption at a behavioural level. In other words, it remains possible that participants never actually achieved abstinence. Another interpretation of our results is that the GM atrophy of insula in AUD groups persists even with the absence of addictive behaviour. Brain atrophy in AUD patients did not reach the equivalent level to controls after abstinence [20]. Based on the evidence in opioid dependence, hypothetically, the GM volume may stay constant once it recovers to normal at a neurological level [39, 40]. Since the GM volume of the insula did not eventually recover to a normal neurological level in the current study, it may be inferred that this insula GM atrophy would continue to deteriorate. As a result, we suggest further studies should consider abstinence in more detail.

Another potential interpretation for the damage in the insula is that the GM atrophy caused “the sense of abstinence” rather than caused by abstinence. The insula plays an important role in the craving of addictive behaviours and it might also facilitate withdrawal symptoms by translating the physiological state of withdrawal into dysphonia [41]. This is only one of many functions of the insula and the GM atrophy in the insula limits its capacity to perform [42], such that the translation from the urge of neurotransmitters into craving is limited. There is empirical evidence in nicotine dependence to support the theory that the GM atrophy in the insula area has been found negatively correlated with tobacco craving [43, 44]. However, this theory has not been directly examined in other types of substance abuse, and the current study observed a comparable pattern, that is, the abstinence duration positively correlated with GM atrophy in the insula. Hypothetically, this GM reduction limited the capacity of the insula and reduced the craving of alcohol consumption to maintain their abstinence.

### Impact of age and gender on GM atrophy

The current study demonstrates a significant negative association between GM in the insula and average the age of the samples, suggesting that any GM deficit would be stronger in older AUD patients. Several previous reviews have illustrated the same interaction between age and GM atrophy [17, 18]. A study by Sullivan et al. [17] assessed the impact of age independently for control and alcoholism groups, and observed that the alcoholism group showed effects of age in the insula, while the control group did not. Additionally, our study was consistent with the results reported by Thayer et al. [18]. In their study, insula deficits caused by alcohol were negatively associated with increased age. However, some evidence points out that GM volume would decrease due to ageing regardless of external interference [45], and consuming alcohol would reinforce the loss of GM to eventually present an alcohol-age interaction [17, 46].

Meta-regression analysis illustrated an increase of GM atrophy in right cingulate gyrus with age at onset in AUD patients. Other studies found that age at onset plays an important role to GM atrophy in AUD patients. For example, those who started drinking before 14 years old were more likely to develop alcohol dependence than those who started drinking after 21 [16]. Moreover, less activation of the cingulate gyrus was also found in adolescents who started drinking after 14 years old compared with no or minimal alcohol users [47]. Our results were different from these two but not contradictory because the previous studies primarily focused on adolescent-onset patients, as the adolescent are more susceptible to the effects of alcohol [15]. Our samples are adult-onset patients, which means the relationship between age at onset and GM atrophy in AUD patients was possibly not absolute linear and requires further investigation.

Additionally, the GM of middle frontal gyrus decreased when the dependence duration increases in AUD patients, which was consistent with the previous analysis that increased alcohol consumption duration will lead to neuronal loss and accelerate with brain atrophy [48, 49]. Our results suggested that further research should include this information as these might have impact on brain structure. Considering that the age at the time of assessment was correlated with age at onset in alcohol studies, the brain region affected by age and age onset might also be affected by the duration of illness, that longer duration was related to higher GM atrophy. However, we found the brain regions are affected by age, age onset and illness duration are different. It is important to point out that the results of age at onset and duration of dependence should be interpreted with cautious, where less than half of the 23 studies had reported the information to be included in the meta-regression analysis, which makes the results cannot be generalised to the whole samples. To address this point, we suggest future studies to report the details of the illness duration.

Finally, the present research revealed a negative association between GM in the left middle frontal gyrus and the gender of the samples, showing that males are more vulnerable to the harmful effects of alcohol on GM than females. Other studies have reported gender differences in GM proportion in intracranial content [50] and age-gender interactions in the hippocampus [45]. Though our study was not consistent with the results reported by Hommer et al. [50], which included a more balanced gender ratio (male = 43; female = 36) in their analysis compared to the current study (male = 658; female = 188). Therefore, the lack of female cases in the present work potentially obscured the impact of gender on GM atrophy.

## Limitation

There were several limitations for the current study. Firstly, the analysed data comprised the coordinates in the published studies rather than the original data which may result in less accurate findings [51]. Secondly, the heterogeneity of methodologies in VBM studies including MRI machines, smooth kernels and corrected level, for example FWER or FDR, might represent a critical factor and couldn’t be controlled for. However, we examine these potential confounders by using quality of the study, which did not affect our results (supplementary material Fig. S3). Thirdly, the current study could not control whether participants had previously smoked, concurrent drinking [52], cardiovascular disease [53]. The studies did not record this information but may contribute to GM thinning in AUD patients. Fourthly, alcohol use disorders and other psychiatric disorders are commonly co-occur co-morbidities, our current results could not be generalised to comorbidity population (Alcohol Use Disorder and Co-Occurring Mental Health Conditions). We suggested that future studies of this field should provide a more detailed comparison between patients with and without comorbidity.

## Conclusions

The current research reported partially consistent results with previous reviews. It reported that GM was lower in the insula, cingulate gyrus, and middle frontal gyrus in the AUD group compared to controls. Moreover, there was lower GM in the insula with increased mean abstinence duration of the samples in the studies. GM atrophy deteriorated more with an increased mean age in our samples, and the males of the samples were reported to be more sensitive to the GM atrophy among AUD patients. The current study included studies which reported their treatments and others did not, making it unable to fairly determine the impact of treatment on GM atrophy. The future research should consider reporting their treatment with more details and placing heterogeneous analysis on the effect of treatment.

## Supplementary Information

**Additional file 1 Fig. S1.** Funnel plots were used to assess publication bias. The funnel plot did not reveal any publication bias as Egger test > 0.05. **Fig. S2.** Meta-regression analysis results. **a** GM in right cingulate gyrus was significantly negatively associated with age of onset in AUD patients. **b** GM in left middle frontal gyrus was significantly negatively associated with duration of illness in AUD patients. Blue color represents GM reduction. **Fig. S3.** Region differences in GM in AUD patients in subgroup meta-analysis of MRI machines (**a**) and smooth kernels (**b)**. Blue color represents GM reduction. **Fig. S4.** Visualize of the samples sizes included in our meta-analysis. **Table S1.** Each study described abstinence duration in AUD patients.

## Data Availability

The data and materials during this study can be made available in this article.

## Abbreviations

GM: grey matter
AUD: alcohol use disorders
MRI: magnetic resonance imaging
VBM: voxel-based morphometry
PFC: prefrontal cortex
ACC: anterior cingulate gyrus
AES-SDM: anisotropic effect size-signed differential mapping
