# Supplementary material for: Lower regional grey matter in alcohol use disorders: evidence from a voxel-based meta-analysis
Source: BMC Psychiatry. 2021 May 11;21:247. doi: 10.1186/s12888-021-03244-9 (PMC8111920; doi:10.1186/s12888-021-03244-9)
Supplement: Supplementary file 1 — Additional file 1 Fig. S1. Funnel plots were used to assess publication bias. The funnel plot did not reveal any publication bias as Egger test > 0.05. Fig. S2. Meta-regression analysis results. a GM in right cingulate gyrus was significantly negatively associated with age of onset in AUD patients. b GM in left middle frontal gyrus was significantly negatively associated with duration of illness in AUD patients. Blue color represents GM reduction. Fig. S3. Region differences in GM in AUD patients in subgroup meta-analysis of MRI machines (a) and smooth kernels (b). Blue color represents GM reduction. Fig. S4. Visualize of the samples sizes included in our meta-analysis. Table S1. Each study described abstinence duration in AUD patients. [file 12888_2021_3244_MOESM1_ESM.docx]

**Supplementary materials**

**Figure Legends**


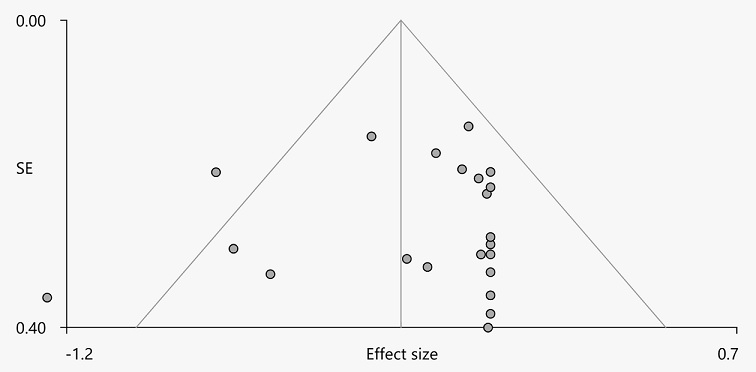


**Figure 1.** Funnel plots were used to assess publication bias. The funnel plot did not reveal any publication bias as Egger test > 0.05

To explore whether the variables of age at onset and duration of dependence change our results, we did the meta-regression analysis for age at onset and duration of dependence in AUD patents. The results as follows: GM in right cingulate gyrus (MNI coordinate: 2, -4, 40; 14 peak voxels; SDM z = -2.965; p = 0.004) was significant negatively correlated with age at onset of the samples (Figure 2a). There was a negative association between the illness duration of the samples and GM in left middle frontal gyrus (MNI coordinate: -28, 44, 30; 4 peak voxels; SDM z = -2.349; p = 0.0039) (Figure 2b). AUD patients’ age were significantly correlated with their age at onset (r= .38, p<.001) and illness duration (r= .35, p<.001), whereas the age at onset of patients was significantly correlated with illness duration (r= .41, p<.001).
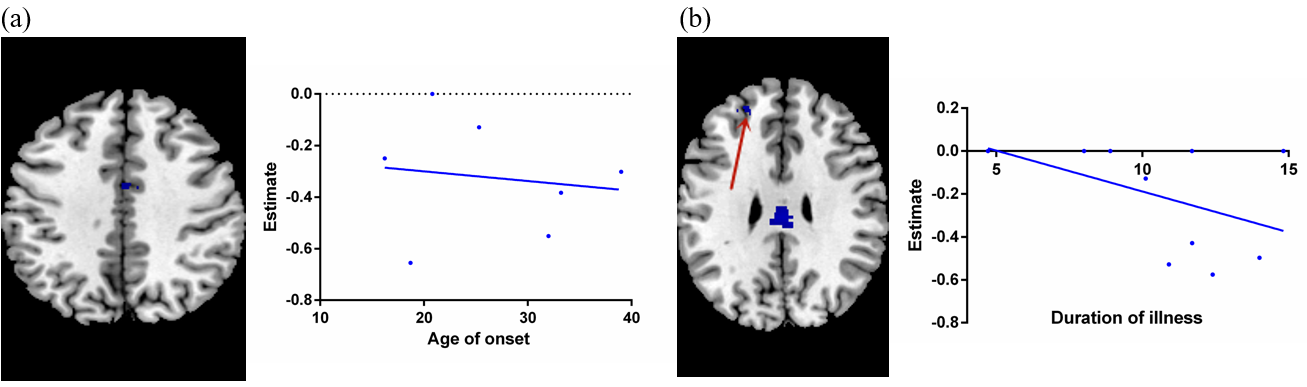


**Figure 2.** Meta-regression analysis results. (a) GM in right cingulate gyrus was significantly negatively associated with age of onset in AUD patients. (b) GM in left middle frontal gyrus was significantly negatively associated with duration of illness in AUD patients. Blue color represents GM reduction.

To assess the critical factors of MRI machines and smooth kernels, we performed subgroup analysis on studies with the 3.0T MRI and the studies with the smooth kernel with 8 mm respectively. The brain regions in left middle frontal gyrus, right insula and the right cingulate gyrus were preserved. the results as follows:
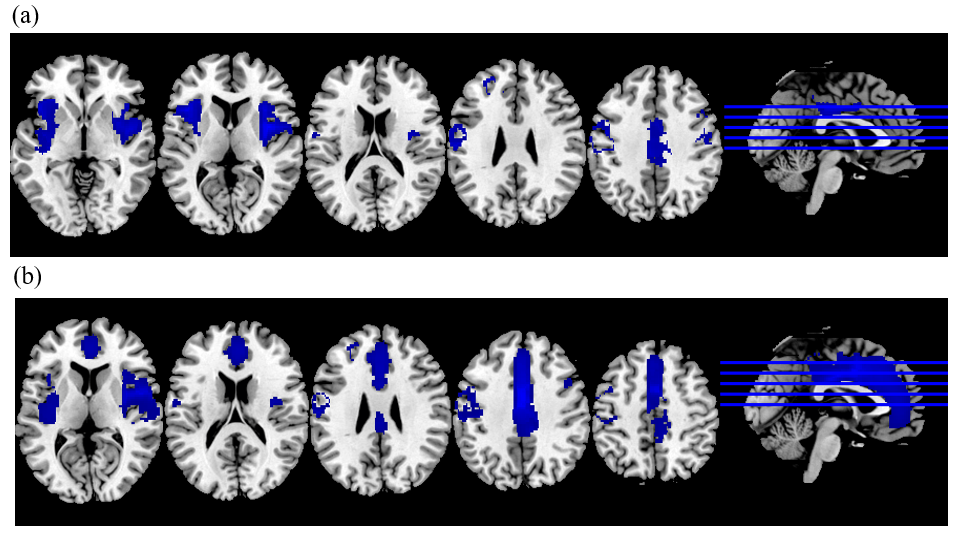


**Figure 3.** Region differences in GM in AUD patients in subgroup meta-analysis of MRI machines(a) and smooth kernels(b). Blue color represents GM reduction.


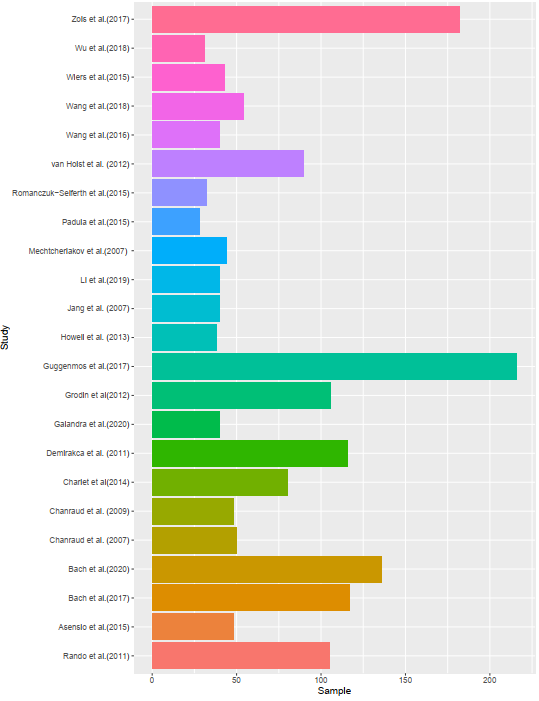


**Figure 4.** Visualize of the samples sizes included in our meta-analysis.

**Table 1 Each study described abstinence duration in AUD patients.**

| Study | AUD patients |  |  |  |  |  |  |  |  |  |  |
| --- | --- | --- | --- | --- | --- | --- | --- | --- | --- | --- | --- |
|  | Abstinence duration |  |  |  |  |  |  |  |  |  |  |
| Jang et al. (2007) | Days of sobriety before the testing(Mean=7.8) |  |  |  |  |  |  |  |  |  |  |
| Mechtcheriakov et al.(2007) | Patients were investigated after alcohol abstinence of at least 10 days | | |  |  |  |  |  |  |  |  |
| Chanraud et al. (2007) | Patients had to have been detoxified for at least 3 weeks and to be abstaining as assessed by biological norms (normal levels of gamma-glutamyl-transferase and normal levels of carbohydrate-deficient transferring | | | | | | | | | |  |
| Chanraud et al. (2009) | Detoxification treatment for at least three weeks, and abstinence as assessed by levels of gammaglutamyl-transferase and carbohydrate-deficient transferrin in normal range | | | | | | | |  |  |  |
| Demirakca et al. (2011) | All patients were studied within the first 5 weeks of detoxification (4 to 37 days after the last alcohol consumption, with a mean of 16.5 ± 7.3 days)  16.5 ± 7.3 days; S1)  (4 to 37 days after the last alcohol consumption, with a mean of  16.5 ± 7.3 days | | |  |  |  |  |  |  |  |  |
| Rando et al.(2011) | Patients underwent a single structural MRI scan after 1 month of alcohol abstinence during their inpatient treatment stay | | | | | |  |  |  |  |  |
| Grodin et al(2012) | Alcoholic subjects were scanned 21.5 ± 5.3 days from admission date and thus had been free of alcohol or other substance use for at least that duration | | | | | | |  |  |  |  |
| van Holst et al. (2012) | To ensure that all participants were detoxified from alcohol, AUD participants had to be fully abstinent for at least two weeks to be included in the study (mean abstinence duration: 18 days), which was assessed by self-report. | | | | | | | | | | |
| Howell et al. (2013) | All participants were asked to refrain from alcohol consumption at least 24 hours before scanning and underwent a urine drug screen and an alcohol breathalyzer test. | | | | | | | |  |  |  |
| Charlet et al(2014) | Alcohol dependent patients were detoxified for 4 to 25 days before MRI(11.4 ± 5.5 days). | | | |  |  |  |  |  |  |  |
| Asensio et al.(2015) | Subjects refrained from alcohol use at least during 3 days prior to the scanning procedure | | | |  |  |  |  |  |  |  |
| Padula et al.(2015) | Alcohol dependence individuals were abstinent from all substances except tobacco for at least 1 month prior to the magnetic resonance imaging (MRI) session. | | | | | | | |  |  |  |
| Romanczuk-Seiferth(2015) | Detoxification took place an average of 42 ± 35 days before the fMRI experiment. | | | |  |  |  |  |  |  |  |
| Wiers et al.(2015) | Abstinence (days) was only mentioned in the table(Mean=48). | |  |  |  |  |  |  |  |  |  |
| Wang et al.(2016) | All patients were abstinent for at least 1 month. | |  |  |  |  |  |  |  |  |  |
| Guggenmos et al.(2017) | Individuals with alcohol dependence had to meet criteria for AD for at least 3 years and had to undergo an inpatient detoxification phase (average duration, ±SEM: 22.8 ± 1 days). | | | | | | | | |  |  |
| Bach et al.(2017) | They had to have completed detoxification successfully (i.e.treatment-if necessary-of withdrawal symptoms with short-acting benzodiazepines or chlormethiazole had to be completed for at least 3 days; i.e. >5× elimination half-life)(Mean=2.5). | | | | | | | | | | |
| Zois et al.(2017) | Alcohol dependent patients had completed medically assisted withdrawal and had stayed abstinent for at least 3 and at most 28 days (11.7 ± 6.6 days). | | | | | | |  |  |  |  |
| Wu et al.(2018) | All patients were detoxified with diazepam(Mean=14) | |  |  |  |  |  |  |  |  |  |
| Wang et al.(2018) | Alcohol dependent patients were interviewed and asked whether they abstained or relapsed by telephone once every two weeks for a period of 3 months after an average of 50.58 days abstinent. | | | | | | | | |  |  |
| Li et al.(2019) | Not mention |  |  |  |  |  |  |  |  |  |  |
| Bach et al.(2020) | The assessment of the patient group took place after a mean abstinence duration of 11.77 days | | | | |  |  |  |  |  |  |
| Galandra et al.(2020) | Patients, recruited during a 28-days alcohol withdrawal treatment, were interviewed to determine their drinking history, including the amount, type and lifetime duration of alcohol use | | | | | | | | |  |  |

AUD = Alcohol abuse disorders.
